# IRRATIONAL FALL RISK APPRAISAL GROUP CHARACTERISTICS IN COMMUNITY-DWELLING OLDER ADULTS

**DOI:** 10.1093/geroni/igad104.2278

**Published:** 2023-12-21

**Authors:** Aleatha Rossler, Ladda Thiamwong, Jeffrey Stout, Victoria Loerzel, Boon Peng Ng

**Affiliations:** UCF, Orlando, Florida, United States; University of Central Florida, Orlando, Florida, United States; University of Central Florida, Orlando, Florida, United States; University of Central Florida, Orlando, Florida, United States; University of Central Florida, Orlando, Florida, United States

## Abstract

Approximately 43% of older adults are affected by fear of falling (FOF). A portion of those affected by FOF have irrational fall risk appraisal (FRA) which means they have a high FOF despite normal balance. This secondary analysis aimed to examine the 18% of the 124 study participants living with irrational FRA from the RO3AG06799-0 data source. Descriptive statistics were used to examine characteristics of older adults with Irrational FRA. Spearman Rho and linear regression were used to evaluate factors (age, sex, race, education, financial status, living situation, fall history, depression symptoms, anxiety, hand grip strength, balance, and fear of falling) associated with Irrational FRA in older adults. Of the 22 participants with irrational FRA, 20 were female, 70% were aged 70 or above (average age 74), and 72% were white (25% Asian, AA, and Hispanic). 77% had anxiety symptoms, with 50% having high anxiety scores, and 32% had depression symptoms. 40% lived alone, 45% had no prior falls, and 70% had low hand grip strength. 23% had inadequate financial means, and 72% had a college education or higher. Statistically significant correlations were found between the FES score and age (p=.044), who the participant lived with (p=.033), and balance (p=.042). Regression analysis showed predictors of FES score in this group to be anxiety (p=.042) and who they live with (p=.024). This study provides valuable insights into the demographic and clinical characteristics of older adults with irrational FRA and highlights the role of anxiety and living status in predicting FOF.

